# Online platform for healthy weight loss in adults with overweight and obesity - the “POEmaS” project: a randomized controlled trial

**DOI:** 10.1186/s12889-018-5882-y

**Published:** 2018-08-01

**Authors:** Alline Maria Beleigoli, Andre Queiroz de Andrade, Maria de Fátima Haueisen Diniz, Roberta Sonia Alvares, Antonio Luiz Ribeiro

**Affiliations:** 1 Internal Medicine Department, Faculty of Medicine, Universidade Federal de Minas Gerais, Av.Alfredo Balena, 190, Belo Horizonte, Minas Gerais CEP 30130-100 Brazil; 2 Telehealth Center of the Hospital das Clínicas of Minas Gerais, Av.Alfredo Balena, 110, Belo Horizonte, Minas Gerais CEP 30130-100 Brazil; 3 Flinders Digital Health Centre, Flinders University, 1284 South Road, Clovelly Park, Adelaide, South Australia 5042 Australia; 4 Department of Medicine, University of Adelaide, North Terrace, Adelaide, South Australia 5000 Australia; 5 Quality Use of Medicines and Pharmacy Research Centre, School of Pharmacy and Medical Sciences, University of South Australia, Adelaide, South Australia 5001 Australia

**Keywords:** Obesity, Overweight, Behaviour change, eHealth, Telemedicine

## Abstract

**Background:**

Obesity is a major health problem in Brazil affecting 19% of Brazilian adults with a rising incidence over the last 10 years. Moreover, low fruit/vegetables consumption and high sweetened beverage intake are major issues. Facing the challenge of universal healthcare access, internet-based programs have the potential to reach a large number of inhabitants, be widely accessible and cost effective. Our aim is to to assess the efficacy of a web-based platform to promote weight loss and diet and physical activity habits change in a Brazilian adult population.

**Methods:**

We designed a three-arm parallel randomized controlled trial including 18–60 years university students or employees with 25 kg/m^2^ minimum body mass index (BMI). Pregnancy, conditions with specific dietary requirements and participation in other weight loss programs are exclusion criteria. Participants are allocated to one of three groups: (1) waitlist with minimal intervention, (2) web-based platform, (3) web-based platform plus online dietician assistance. Assessors are blinded. Weight and BMI loss are the primary outcomes. Diet and physical activity behaviours, health perception and online activity features are secondary outcomes. The intervention comprises recommendations of diet and physical activity habits tailored to the Brazilian population and principles of behaviour change. The web-based platform has online social network and gamification features. Analysis will be on an intention-to-treat basis at 12 and 24 weeks after baseline. Differences in weight loss between groups will be performed by analysis of covariate. Linear regression will be used to assess whether treatment group allocation is an independent predictor of weight loss. The study was approved by the Federal University of Minas Gerais (UFMG) Ethics Research Committee. All participants signed an informed consent form prior to recruitment.

**Discussion:**

We present the study protocol of a three arm parallel randomized controlled trial which aims to test the efficacy of an online platform to promote weight loss for adults with overweight and obesity. We anticipate that the adoption of healthy lifestyle habits and weight loss will be more important in participants randomized to the online platform group.

**Trial registration:**

NCT03435445 on February 16th, 2018.

## Background

According to the World Health Organization, more than 600 million people ≥18 years-old were obese (Body mass index- BMI ≥ 30 kg/m^2^) in 2014. Obesity has been associated with a serious burden due to high morbidity, mortality and impact in quality of life. The escalating obesity prevalence is a well-known phenomenon in developing countries. Also in Brazil, following the population and nutritional transition, obesity has increased 60% over the last 10 years among adults. According to a 2016 telephone-based survey which interviewed over 53,000 adults in city capitals, 50% of the adults have weight excess (BMI ≥ 25 kg/m^2^) and obesity affects 19% of the Brazilian adult population. Moreover, 16% of the population takes sweetened beverages five or more days per week and only 35% of the population eats fruits and vegetables regularly [1]. Given this appalling statistics, the Ministry of Health established sweetened beverages and fruits and vegetables consumption as pivotal strategies in the aim of stabilizing the growth of obesity until 2019 [2]. Hence, developing strategies to target obesity and unhealthy lifestyle habits is a national priority.

Access to healthcare, particularly in remote and underserved areas, is a challenge around the world. In this context, eHealth, which is defined as the use of information and communication technologies for health and, particularly, internet-based programs have the potential to reach a large number of people, be widely accessible and cost effective [3]. A 24 h/7d accessibility, affordability, anonymity and opportunity are additional advantages of web-based weight loss programs in comparison to traditional face-to-face interventions [4]. Despite all these potential benefits, results have been heterogeneous in regards to weight loss results [5]. This may be due in part to the low long-term adherence and high dropout rates observed in interventions that demand behavior change regardless being face to face or technology-based [6, 7]. Moreover, the use of mixed behavioral and the wide range of technological strategies currently available, such as mobile phones, patient monitoring devices, personal digital assistants (PDAs) and wireless devices and telehealth, might explain these diverse outcomes and makes it difficult to understand which components are associated with better results. Tailoring technology tools to particular population characteristics, such as age, gender, socioeconomic level, as well as health and technology literacy probably enhances adherence and motivation, which are key points to the success of weight loss programs [7].

Our aim is to report the study protocol of a randomized controlled trial (RCT) which investigates the efficacy of an online weight loss program named “POEmaS” (acrostic for online platform for healthy weight loss in Portuguese) on weight loss of Brazilian adults with overweight and obesity up to six months. We hypothesized that the web-based intervention with dietary recommendations specific to the Brazilian population either without or with individualized support is associated with greater weight loss and greater adoption of healthy lifestyle habits than a minimal intervention.

## Methods

### Design

This is a three-arm parallel randomized controlled trial which recruited adults (18–60 years) participants classified as overweight or obese (body mass index – BMI > =25 kg/m^2^) and who had the intention to lose weight.

### Participants and recruitment

Students (current and past) and staff of the Federal University of Minas Gerais (UFMG), Belo Horizonte, in the southern Brazil, were recruited by physical and online advertising (flyers, University website, UFMG mailing list) for one month from the 25th September to 24th October, 2017. They were invited to access the project website www.poemasufmg.com.br, where they can watch a video with details of the project. Interested participants can join the project online and create an account profile after checking for the inclusion and exclusion criteria (Table 1) and signing the informed consent form. During the recruitment phase, participants could invite friends, colleagues and acquaintances to whom they thought the platform might be helpful by informing their email.

**Table 1:** Inclusion and exclusion criteria for recruitment into “POEmaS (Online Platform for healthy weight loss)” Randomized Clinical Trial

Inclusion criteria	Exclusion criteria
Age between 18 and 60 years	Pregnancy
BMI^a^ > = 25 kg/m2	Self-report of comorbidities that demand specific dietary or physical activity recommendations- diabetes, heart failure, coronary artery disease, kidney disease, hepatic disease, cancer, phenylketonuria, celiac disease, food allergies, bariatric surgery history
Weight report at entry	Participation in any other weight loss program
Intention to lose weight by changes in lifestyle habits	
Internet access	

^a^*BMI* Body mass index

### Randomization and allocation

Once informed consent was obtained, participants were randomly allocated to one of the three groups using a stratified randomized block design. Participants were stratified by gender and category of body mass index (25 to < 30 or ≥ 30 kg/m^2^) using blocks of variable length (either 3 or 6).

**Fig. 1:**
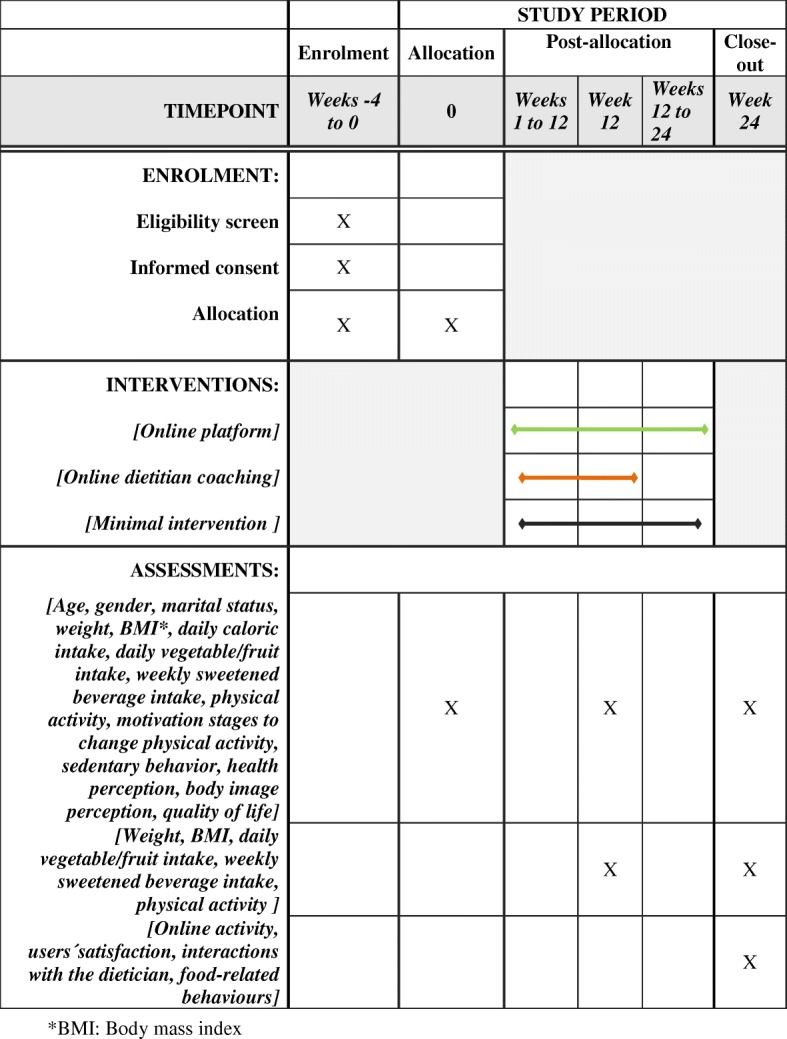
Details on enrollment, allocation, intervention and assessments according to SPIRIT guidelines

Participants were initially allocated to one of three groups as shown in Fig. 1:

1. Group 1, control group which is submitted to an initial minimal intervention for 24 weeks and subsequently will have access to all the platform functionalities;
2. Group 2 will follow a standard weight loss program delivered by the website platform for 24 weeks;
3. Group 3 followed the standard weight loss program enhanced by personalized feedback by a nutritionist based on lifestyle habits reported in the platform for 12 weeks and the standard weight loss program for the following 12 weeks.

Subsequently, subjects received an automatic email with detailed information on the groups they were allocated to. Researchers were blinded to participants´ allocation.

### Outcome measures

All outcomes will be assessed at baseline, 12 and 24 weeks afterwards. Outcome assessors will be blinded to group allocation. Weight and BMI change are the primary outcomes of the study and will be self-reported by the participants. Secondary outcomes will be changes in dietary habits, food-related behaviors, physical activity habits, sedentary behaviors, quality of life, health perception, online activity and users´ satisfaction, as detailed in Fig. 1.

### Sample size

Based on a 90% power to detect a significant difference of 4 kg weight loss between groups, assuming the SD of weight is 6.0 and using a two-sided significance level of 0.05, and a 40% attrition rate, a sample size of 90 participants was calculated for each group [7].

### Interventions

#### Weight-loss and behavior change interventions in each group

The interventions will be delivered online for 24 weeks, with new program content distributed to the user weekly for Groups 2 and 3. The content of dietary and physical activity recommendations was based on Brazilian and international evidence-based guidelines [8]. Dietary recommendations focused on particular Brazilian habits. Physical activity recommendations focused on increasing time of leisure exercise and decreasing sitting time.

In group 1, subjects have access to four videos with information on health consequences of excessive weight, dietary and physical activity weight loss recommendations, as well as strategies to behavior change. They also received an e-book with lifestyle habits and behavioral recommendations to lose weight. Access to the study website will be limited for the first 24 weeks and they have been asked not to undertake any other weight-loss program during this time. They will be invited to report their weight, dietary and physical activity habits at 12 and 24 weeks after baseline by email. Afterwards, they will gain access to the standard online platform.

Group 2 has access to the study website program for 24 weeks. The platform used in this study was adapted from a commercial product (Cybergia Tecnologia em Sáude® Inc.), which runs various Internet-based health programs in Brazil. The platform is cloud- based progressive, web-app developed and can be accessed in desktop and smartphone environments. It has three areas: 1) Main area, presenting weekly updated tasks such as content (video, articles), suggested dietary and physical activities and questionnaires; 2) Self-monitoring area, containing a food diary, functionalities for activity self-report and charts; 3) Social area, containing a private social network where users can share and react to photos and posts. Connecting these areas, the platform presents gamification mechanics, such as weekly points (connected to completing tasks and interacting with other participants) and social challenges, which invite users to complete one-time specific tasks and share their accomplishments. Interaction with the project’s team was restricted to public posts and selected comments on the social network.

Group 3 has access to this online program plus weekly individual chat in the platform with a dietitian. At these times, they have the opportunity to discuss and manage individual issues.

**Fig. 2:**
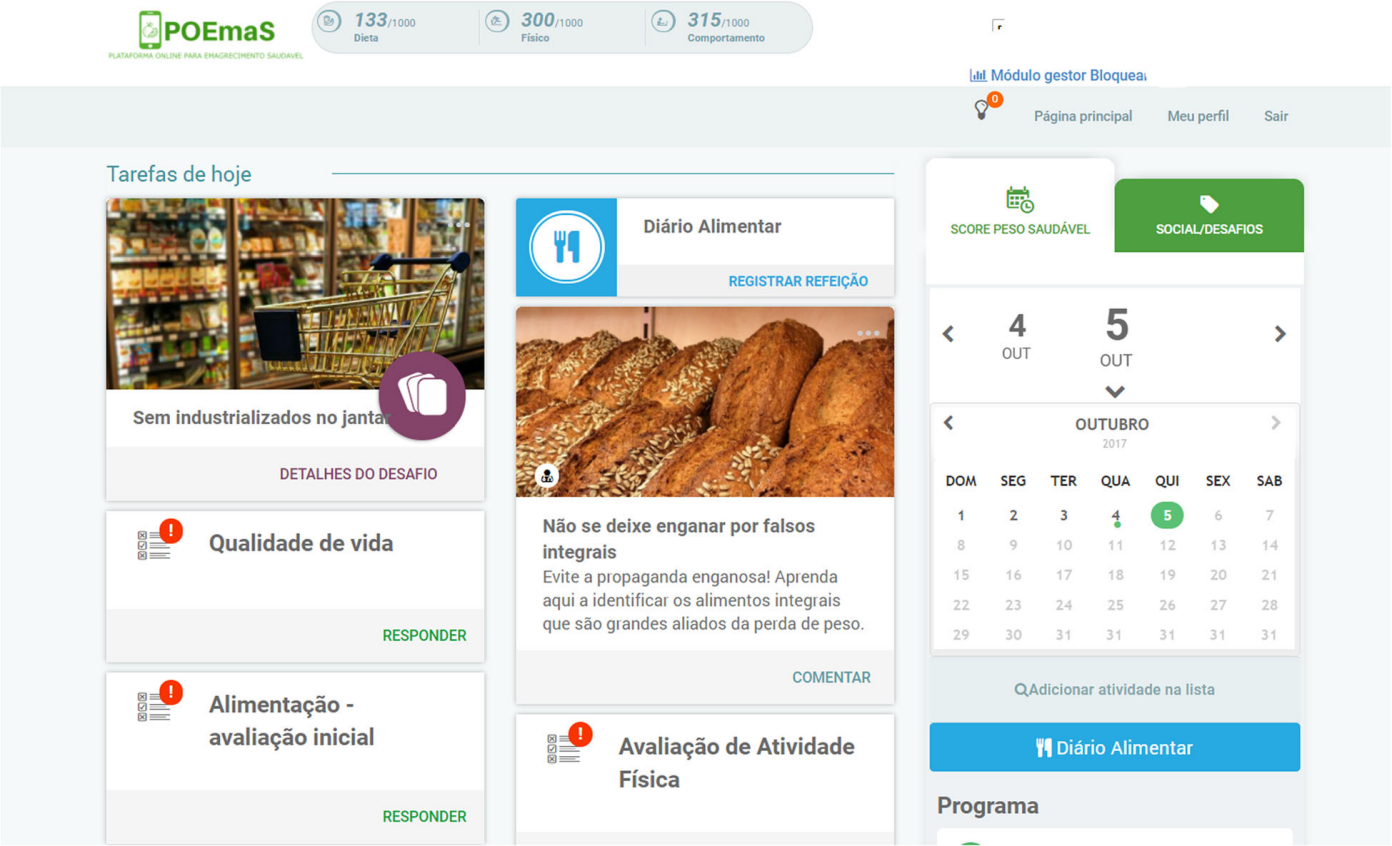
Print screen of the main page of the “POEmaS” online platform in Portuguese (extracted from the web platform used in the study)

Print screens of the main page of the platform can be seen in Fig. 2.

#### Quality control

Several procedures will be employed to optimize the quality of the study and maximize internal validity and reliability of the program delivery and outcome assessments. Assessors of the main outcome measures are blinded to the participants’ group allocation. Assessment protocols and orientations to participants were standardized in written documents. Validation of self-reported weight and height will be performed by correlation to measurements for a random sample of 10% of the study population. Personnel will be trained on anthropometry procedures prior to the assessment. Regular team meetings between the clinical and information technology teams, in order to align strategies, are planned.

#### Measurements and instruments

##### Demographic data

Data on gender, education, marital status and place of work will be collected at baseline. Questionnaires to obtain current medical conditions and medications will be completed at baseline and 24 weeks.

##### Anthropometry

Weight and height will be self-reported by the participants and BMI will be calculated by the formula weight in kg/ (height*height) in m^2^ [9]. For the random 10% sample selected for additional measurements, weight will measured in light clothing, without shoes on a Welmy® analogic scale with stadiometer. For this sample, waist circumference will be measured at the mid-level between the last rib and the iliac crest using an inelastic tape and body composition (body fat percentage, free fat mass percentage) will be measured by tetrapolar bioelectrical impedance using Sanny® Bioimpedance [10].

##### Dietary intake and food-related behavior

For groups 2 and 3, calculation of the total daily caloric intake and dietary nutrients will be performed by an automatic dietary calculator which was based on food nutrition composition tables for the Brazilian population [11].

Questions about the total number of daily serves of fruit and vegetables, and whole carbohydrates sources, as well as weekly serves of sweetened beverages and snack foods will be applied. Moreover, food-related behaviors, including number of days taking breakfast, number of daily meals, frequency of take-away food consumption and eating while watching television will be assessed by questionnaires.

##### Eating behavior

The random sample selected to anthropometry will have cognitive and behavioral components of eating assessed across three scales of cognitive restraint, uncontrolled eating and emotional eating by the Brazilian version of the Three Factor Eating Questionnaire-R21 (TFEQ-R21) [12].

##### Physical activity and sedentary behavior

- The short-form of the International Physical Activity Questionnaire (IPAQ) will be used to estimate total MET-minutes/week and to classify participants into either high, medium or low physical activity categories according to the IPAQ Scoring Protocol [13].
- The daily screen-time (i.e., in front of TV, computers, video games, smartphones) and the daily sitting time will be used to assess sedentary behavior.
- Pedometers: the number of steps will be measured by pedometer in the sample randomly selected to anthropometry.

##### Health perception

The Brazilian version of the *12-item Short-Form Health Survey – Version 2*, (QualityMetric Incorporated, Lincoln, RI, USA) which is a self-reported measure of health-related quality of life (HRQoL) designed to investigate multidimensional aspects of physical and mental health for the general population and those with chronic diseases, will be applied to all participants [14].

Self-rated health will be assessed by one question derived from the Short-Form-36(SF-36)- “In general, you say your health is…” with “excellent”, “very good”, “good”, “regular” or “bad” as alternative answers [15].

Body image perception and satisfaction will be assessed by a scale in which the participant can choose his current and ideal silhouette from 15 options, according to gender [16].

##### Online activity and satisfaction with the weight loss program

Self-monitoring entries, posts and comments in forums and chats will be collected and stored in the platform database and exported as CSV files. For Group 3, we also analyzed the number of interactions with the nutrition specialist. To assess users´ satisfaction with the weight loss program and with the web-based platform we will develop and apply a specific questionnaire.

#### Data analysis

Analysis will be on an intention-to-treat basis. Outcomes will be analyzed at two time-points (12 and 24 weeks). Analysis of covariance will be used to test for differences in weight loss between groups at each time point. The models will be fitted using linear regression with weight as the outcome variable, treatment group as the predictor variable of interest and weight at baseline included as a covariate. Age, gender, dietary intake (kilocalories) and physical activity at baseline will enter the model for adjustment. Statistical significance of the primary efficacy analysis (at 12 and 24 weeks) will be based on Hochberg’s multiple testing procedure with the family wise error rate for each time point held at 2.5%. Secondary hypothesis tests will be performed using a 2-sided 5% significance level [17–20].

## Discussion

We applied the Behaviour Change Wheel framework to develop the behaviour program delivered by the web platform [21]. The program includes the following elements: setting realistic goals; self-monitoring of weight, body measurements, exercise and diet; controlling of stimuli that activate eating; eating style; cognitive restructuring; dealing with expectations; modelling, social support and personalization. The platform features, according to each behavior strategy element, are displayed in Table 2.

**Table 2:** Behavior strategies and corresponding platform features

Behavioral strategy	Platform features
Knowledge/empowerment	Short educational texts on healthy dietary, physical activity and sleeping habits Short texts on behavioral strategies to enhance self-efficacy Low-calorie menu suggestions Recipes Metabolic resting energy expenditure calculations based on age and activity level Physical activity tips according to exercise preferences
Goal setting	Weight loss and calorie intake goal setting oriented by realistic goals Graphical display of changes in weight, BMI and waist Individualized daily calorie targets to facilitate 0.5–1 kg weight loss per week, based on participants weight, height and activity level
Outcomes expectations	Automated recommendations on realistic goals linked to self-monitoring features
Self-monitoring	Searchable online food diary to facilitate entry of food data Automated weekly reminders for entering weight and specific habits considered ‘weak points’ by the user Calculations summaries of energy balance and nutrition referenced to recommended nutrient targets and individualized goals
Modeling	Recipes Promotion of users´ successful weight loss/behavior change reports on the online social network Scenarios with clinical cases to educate users on how to deal with weight loss issues based on real situations
Social support	Online forums with participation of the users and of the project team for informational and emotional support, as well as problem-solving assistance Challenges, which can be shared in the platform social network
Personalization	Definitions of individual ‘weak points’ Automated, computer-generated, personalized feedback based on their diet and physical activity diaries, ‘weak points’ and on the use of website features

## Abbreviations

BMI: Body Mass Index
CSV: Common Separated Value
HRQoL: Health-Related Quality of Life
IPAQ: International Physical Activity Questionnaire
MET: Metabolic Equivalent
PDAs: Personal Digital Assistants
RCT: Randomized Controlled Trial
TFEQ-R21: Three Factor Eating Questionnaire-R21
UFMG: Federal University of Minas Gerais
